# Chitinase-1 Activity in Serum of Cats with FIP

**DOI:** 10.3390/v15091815

**Published:** 2023-08-25

**Authors:** Angelica Stranieri, Gabriela Ávila Morales, Laura Brusasco, Saverio Paltrinieri

**Affiliations:** Department of Veterinary Medicine and Animal Science, University of Milan, 26900 Lodi, Italy; angelica.stranieri@unimi.it (A.S.); gby31193@gmail.com (G.Á.M.); laura.brusasco@gmail.com (L.B.)

**Keywords:** CHIT1, inflammation, feline infectious peritonitis, macrophages

## Abstract

Background: Chitotriosidase (chitinase 1 or CHIT1) is secreted by activated macrophages. Macrophages are involved in the pathogenesis of feline infectious peritonitis (FIP). No reports on CHIT1 activity in cats with FIP are available. Objective: To preliminarily investigate the possible changes in serum CHIT1 activity in cats with FIP. Methods: CHIT1 activity was measured in serum samples from clinically healthy cats (n = 17), cats with FIP (n = 19) and cats with diseases potentially characterized by macrophage activation (n = 20), after a preliminary assessment of the imprecision and linearity of the method. Results: The highest CHIT1 activity was found in cats with FIP, followed by sick cats and clinically healthy cats. The magnitude of the differences between groups was higher than the intra- and inter-assay imprecision of the method (<5% and >57%, respectively). Based on receiver operating characteristic (ROC) curves, CHIT1 may differentiate sick from clinically healthy cats and, to a lesser extent, cats with FIP from cats without FIP. Conclusions: CHIT1 activity may identify sick cats and, within the appropriate clinical context, cats with FIP, although larger and more standardized studies, coupled with additional information on analytical performances of the method, are required to fully explore the diagnostic or prognostic potential of this test for FIP.

## 1. Introduction

Chitinases are a class of evolutionary conserved enzymes that catalyze the hydrolysis of chitin, one of the most abundant biopolymers in nature and a structural component of arthropods, crustaceans, mites, fungi and nematodes [1,2]. Chitinases are present in insects, plants, bacteria, fungi and mammals, playing a role in digestion, pathogenicity, arthropod molting, immunity and defense [2]. Humans express two chitinases, chitotriosidase (chitinase 1 or CHIT1) and acidic mammalian chitinase (AMCase), both endochitinases with hydrolyzing activity. In addition, humans also express several chitinase like proteins (CLPs) that are catalytically inactive [2]. CHIT1 was the first to be discovered, and it was detected in macrophages of patients affected by Gaucher’s disease, as it is secreted by abnormal lipid-laden macrophages that develop in the tissues of people affected by this disease [3]. CHIT1, in fact, is secreted by activated macrophages and also expressed in monocyte-derived cells (e.g., Kupffer cells, osteoclasts, dendritic cells), as well as by activated neutrophils [1,4]. It appears that Toll-like receptor (TLR) signaling induces CHIT1 production in neutrophils, while in macrophages it is induced by nucleotide-binding oligomerization domain-containing protein 2 (NOD-2) signaling [5]. Since its discovery, increases in CHIT1 have been described in other lysosomal diseases such as Niemann–Pick disease, but also in sarcoidosis, b-thalassemia, multiple sclerosis, atherosclerosis, Alzheimer’s disease and in parasitic infections [6,7]. Even though the exact mechanisms of the increase in CHIT1 in these conditions is not completely understood, it is widely accepted that this enzyme has an important role in innate immunity, as it is upregulated in response to inflammatory stimuli by neutrophils and macrophages. Moreover, CHIT1 seems to have an additional role during Th2-driven immune response, since monocytes treated with IL-4 significantly increase mRNA CHIT1 expression [6]. To our knowledge, no reports on CHIT1 activity in cats are available and methods to measure CHIT1 activity in feline serum have never been validated. The availability of information about the activity of this enzyme in serum may be useful to study, diagnose and stage diseases that are characterized by a strong activation of innate immunity and especially of macrophages. Among these, feline infectious peritonitis (FIP), a disease of cats sustained by the feline coronavirus (FCoV), may benefit from such assay since the activation of macrophages plays a pivotal role in the development of FIP [8]. The FCoV is widespread in feline populations, colonizes the intestine and may be transported into the bloodstream by the cells of the monocyte-macrophage system without inducing clinical disease, except for mild enteritis. Mutated FCoV variants may replicate within macrophages and colonize various tissues or organs, where, depending on the type and magnitude of the immune responses of the host, they may induce FIP. In particular, the formation of pyogranulomatous lesions, characterized by type IV hypersensitivity reactions, is typical of the so-called dry (or non-effusive) form of FIP, while a systemic vasculitis sustained by a type III hypersensitivity reaction is typical of the so called wet (or effusive) FIP [9]. In both dry and wet FIP, activated macrophages are found in lesions [10], and it may be thus expected that high amounts of CHIT1 are produced by activated cells and released in serum.

Therefore, the aim of this study was to preliminarily assess the ability of serum CHIT1 activity, measured through a commercially available fluorimetric method, in differentiating sick from clinically healthy cats and, among sick cats, between cats with FIP and cats with diseases other than FIP.

## 2. Materials and Methods

### 2.1. Caseload

This was a retrospective study on serum samples selected from our archives on the basis of the final diagnosis. Specifically, the study was carried out on samples collected at the Veterinary Teaching Hospital (VTH) of the University of Milan from patient cats referred to the VTH for routine diagnostic or wellness visits. In all cases, blood samples collected for diagnostic purposes during the visits were placed in plain tubes and immediately transported to the diagnostic laboratory to be centrifuged (2500× *g* for 10 min) and processed for routine clinical chemistry. The leftover serum was then frozen at −20 °C to be included in this study, and stored for a maximum of 18 months until analysis. Data regarding routine hematology performed within the laboratory workup were also reviewed and the number of neutrophils and monocytes in blood was recorded.

Cats were grouped as follows:

- Clinically healthy cats (n = 17): cats which did not present any clinical or laboratory alteration during routine wellness or check-up visits.
- Cats with FIP (n = 19) diagnosed based on anamnesis, clinical presentation and typical clinicopathological and/or pathological alterations on serum, effusions and/or tissues.
- Cats with diseases other than FIP (n = 20). These diseases were diagnosed by an integrated approach, variable depending on the clinical presentation.

Details on signalment, clinical or laboratory findings, including the diagnostic approach followed in the different groups and on the final diagnoses, are reported in Table 1, which also reports the results about neutrophil and monocyte counts in blood.

Statistical analysis (see below) revealed a significant difference among the ages of cats included in the three groups (*p* = 0.002), with higher values in cats without FIP, compared to both cats with FIP and clinically healthy cats (*p* < 0.001 and *p* = 0.008, respectively).

### 2.2. Chitinase Assay

Chitinase activity was measured using a commercially available kit (Chitinase Assay Kit, cat n° CS1030, Sigma-Aldrich, St. Louis, MO, USA) following the manufacturer’s instructions and the procedure already described in a previous study in people [13]. The assay was based on the hydrolysis of chitin to generate 4-methylumbelliferone (4MU), which, once ionized at an alkaline pH, can be measured using a fluorometer. Briefly, wells of black 96-well plates were filled with the substrate working solution, containing dimethyl sulfoxide (DMSO) and 4-Methylumbelliferyl N-acetyl-β-D-glucosaminide diluted in the assay buffer included in the kit, and with 5 µL of samples. The standard solutions included in the kit were added in the plate wells in duplicate. Additionally, two wells were used, respectively, as a “blank well” (filled with 95 µL of working solution and 5 µL of substrate solution) and as a “positive control” well (filled with 95 µL of working solution and 5 µL of the chitinase control enzyme included in the kit). The plates were incubated at 37 °C for 30 min and then the reaction was stopped with 200 µL of a stop solution containing sodium carbonate (4.23%). The plates were then read with the fluorometer Fluoroskan Ascent (Thermo Fisher Scientific, Waltham, MA, USA) using an excitation wavelength of 355 nm and an emission wavelength of 485 nm.

Two sequential sessions of ELISA testing were run on the whole caseload using the same sera, in order to assess the inter-assay imprecision of the method.

Moreover, in a separate session of tests, intra-assay imprecision and accuracy by linearity under dilution were evaluated; to this aim, two approaches were followed: 30 samples randomly selected from the caseload were run in duplicate. Additionally, the 5 serum samples characterized by the highest fluorescence intensity (FI) and the 5 sera characterized by the lowest FI were used to create a “high CHIT1 pool” and a “low CHIT1 pool”, respectively. Each pool was used to fill 5 wells of the plate to further analyze the intra-assay repeatability. The high CHIT1 pool was used to evaluate the accuracy using a linearity under dilution (LUD) test by serially diluting the pool with distilled water to final concentrations of 100%, 80%, 60%, 40% and 20% before testing.

### 2.3. Statistical Analysis

Intra-assay and inter-assay imprecision were calculated using the results of repeated testing described above and were expressed in terms of coefficient of variation (CV), which was calculated using the formula CV = standard deviation/mean × 100.

In the linearity under dilution test, the correlation between the percentage of recovery compared with expected values of LUD test was assessed using a least square regression test. The acceptability of the analytical performance was evaluated using published data on the performance of other assays (state of the art) as a benchmark, as recommended when other methods to assess the clinical demand of acceptability are not available [14].

Results obtained in each session from the three groups of cats (clinically healthy, affected by FIP and affected by diseases other than FIP) were compared to each other using a non-parametric ANOVA test for independent data (Kruskall–Wallis test), followed by a Steel–Dwass test as a post hoc test. The U Mann–Whitney test was used to compare the results obtained in cats with wet or dry FIP. The Kruskall–Wallis test and the Steel–Dwass test were also used to compare the age of the cats enrolled in the three different groups, as well as the number of neutrophils and monocytes in blood. The possible correlation between CHIT1 activity and the number of neutrophils and monocytes in blood was assessed using the Spearmann correlation test.

The assessment of the diagnostic relevance of CHIT1 activity in identifying sick cats (i.e., cats with FIP or with diseases other than FIP) was assessed by calculating, for each test session and for each point value recorded in the study, the number of true positive (TP = sick cats with CHIT1 activity higher than the point value), false positive (FP = healthy cats with CHIT1 activity higher than the point value), true negative (TN = healthy cats with CHIT1 activity higher than the point value) and false negative (FN = sick cats with low PON1 activity higher than the point value) results. Based on the number of TP, FP, TN and FN, sensitivity (Sens) and specificity (Spec) were calculated using standard formulae for all the point values recorded in the study [15]. Using Sens and Spec, the positive likelihood ratio (LR+) was calculated using the formula LR+ = Sens/(1 − Spec), the negative likelihood ratio (LR−) using the formula LR− = (1 − Sens)/Spec, and receiver operating characteristic curves (ROC curves) were designed by plotting Sens vs. (1 − Spec). The area under the curve (AUC) of each ROC curve was then calculated. The calculation of Sens, Spec, LR+ and LR− and ROC curve analysis was then repeated to assess the discriminating power of CHIT1 activity for cats with FIP, grouping clinically healthy cats and cats with diseases other than FIP in a single group of “non FIP cats” and considering as TP and TN the cats with FIP that had CHIT1 activity, respectively, higher or lower than each point value, and FP or TN the non-FIP that had CHIT1 activity, respectively, higher or lower than each point value.

## 3. Results

### 3.1. Analytical Performances of the Method

The intra-assay CVs of the high CHIT1 pool and of the low CHIT1 pool were 7.0% and 5.2%, respectively. However, one outlier was present in repeated readings of the high CHIT1 pool and after the removal of this outlier the CV decreased to 0.6%. The mean and median intra-assay CVs recorded in duplicate readings of 34 serum samples were 7.33 ± 6.8% and 4.8%, respectively.

The mean and median CVs calculated based on the results of the two sessions of tests were 29.7% ± 24.8% and 22.2%, respectively.

The linearity under dilution test revealed an excellent correlation between observed and expected values (*p* < 0.001, rs = 0.996).

### 3.2. Group Comparison

**Figure 1:**
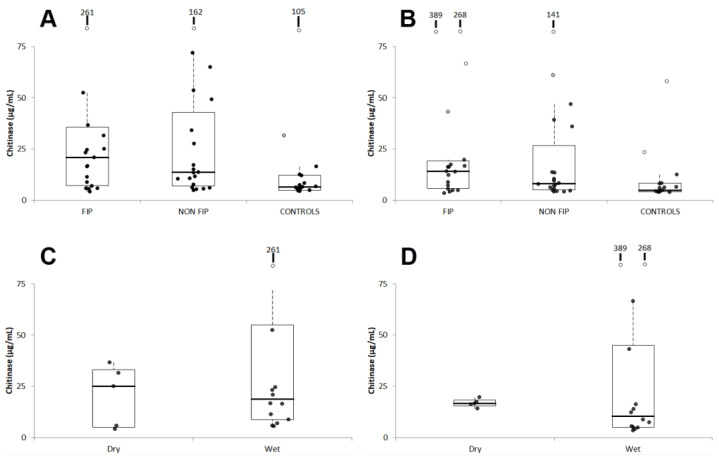
Distribution of results recorded in session 1 (**A**,**C**) or 2 (**B**,**D**) in the different groups of cats or in cats grouped according to the type of FIP. The boxes indicate the I-III interquartile range (IQR); horizontal lines indicate the median value. Vertical lines extend until the last value not classifiable as an outlier. Black circles indicate the results not classifiable as outliers; gray circles and open circles indicate, respectively, the near outliers (values higher than the III quartile + 1.5 × IQR) and the far outliers (values higher than the III quartile + 3.0 × IQR). Far outliers exceeding the scalebar are reported on the top of each graph.

In both the test sessions (Figure 1, Table 2), the highest chitinase activity was found in cats with FIP, followed by cats with diseases other than FIP and by clinically healthy cats. However, compared with clinically healthy cats, the chitinase activity was significantly higher in cats with FIP, but not in cats with diseases other than FIP, and no significant differences between the two groups of sick cats were found.

Moreover, despite a few cats with effusive FIP sometimes having very high values in both test sessions, (Figure 1), significant differences between cats with wet or dry FIP were never detected (Table 3).

The neutrophil counts of sick cats (12.5 ± 11.88 cells/×10³ µL; median: 8.64 cells/×10³ µL) were significantly higher (*p* = 0.021) than those of healthy cats (data reported in Table 1), and a significant difference (*p* = 0.031) was also found when the three groups (healthy, FIP, non-FIP) were compared to each other. However, significant differences in neutrophil counts were found only between non-FIP cats (*p* = 0.007), and not between FIP cats and healthy cats (*p* = 0.190) or between non-FIP cats and FIP cats (*p* = 0.235).

No significant differences were found for monocyte counts, either in the comparison of healthy cats with the whole group of sick cats (0.55 ± 0.78; 0.27) (*p* = 0.812) or in the comparison of the three groups (*p* = 0.301).

No correlations were found between CHIT1 activity and neutrophil or monocyte counts either in the first session of tests (*p* = 0.267 for neutrophils; *p* = 0.472 for monocytes) or in the second session of tests (*p* = 0.208 for neutrophils; *p* = 0.812 for monocytes).

**Figure 2:**
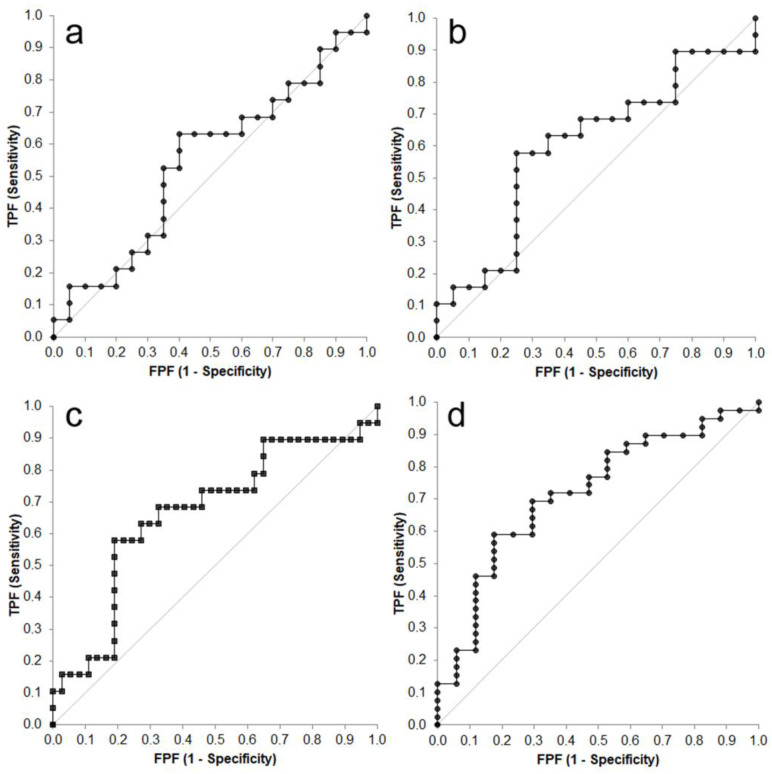
Receiver operating characteristic (ROC) curves of chitinase 1 (CHIT1) designed to differentiate sick from healthy cats ((**a**) = session 1; (**b**) = session 2) or FIP cats from cats without FIP ((**c**) = session 1; (**d**) = session 2). The gray line indicates the line of no discrimination; TPF = true positive frequency; FPF = false positive frequency.

The ROC curve analysis is reported in Figure 2.

This analysis demonstrated that chitinase activity had a good discriminating power to differentiate sick cats (i.e., cats with FIP and with diseases other than FIP) from clinically healthy cats: the AUC was significantly different from the line of no discrimination in both the test sessions (*p* = 0.002 and *p* = 0.004, respectively). Conversely, the discriminating power to differentiate cats with FIP from cats without FIP (i.e., cats with diseases other than FIP and controls) was not significant in one session of tests (*p* = 0.087) and had a weak statistical significance in the other (*p* = 0.044), and the AUCs were not excellent (Table 4).

As shown in the table, the Youden’s index was similar between the test sessions, but in all the cases values of sensitivity and specificity corresponding to the highest Youden index were moderate. As expected, the values of CHIT1 activity that may differentiate sick cats from clinically healthy cats were higher than those that differentiate cats with FIP from cats without FIP, and CHIT1 activity had an absolute specificity to diagnose FIP only at very high values. Moreover, in both clinical settings (sick vs. healthy and FIP vs. non-FIP), threshold values were different in the two sessions of tests.

## 4. Discussion

Based on the current knowledge on its pathophysiology, CHIT1 may also be a powerful biomarker of diseases in cats characterized by an intense recruitment and activation of macrophages, such as FIP. Therefore, we designed this study to preliminarily assess its potential utility in the diagnostic approach to feline diseases, with special emphasis on the diagnosis of FIP.

The results of this study demonstrate that the intra-assay imprecision of the method is low, although some individual readings may be excessively high. However, even in the presence of these outliers, the CVs were comparable with those considered as acceptable for many tests commonly used in clinical pathology [16]. As expected, the inter-assay imprecision was higher than the intra-assay imprecision. This commonly occurs in clinico-pathological tests [17] as a consequence of several possible factors. Among these, the storage of samples and reagents between different sessions of tests may have played a relevant role in increasing the inter-assay imprecision. The possible influence of storage time and temperature and of cycles of freezing and thawing of samples has not been investigated in this study but deserves to be included in future studies, either to better interpret the results of samples processed in different sessions of tests, or to provide practical information on sample handling for diagnostic purposes.

In the absence of a gold standard test to measure CHIT1 activity in feline serum, or of reference control material to assess accuracy through spiking recovery tests, accuracy was estimated indirectly through a linearity under dilution test. This approach revealed that in each test session in which the LUD test was performed, the linearity was good to excellent, suggesting that the test is accurate.

Altogether, these results indicate that the fluorimetric test to measure the activity of CHIT1 in feline serum is precise and accurate, although the inter-assay variability between different sessions of tests may be high. Information about storage stability, as well as other information usually included in validation studies such as the effect of interferents and the possible sources of intraindividual variability [18,19], needs to be assessed in future studies. The preliminary validation included in this study was mostly focused on precision and accuracy in order to produce information on how the possible differences between pathologic groups can be interpreted.

From this perspective, the results recorded in the different groups of cats confirm that CHIT1 activity may be a good marker of disease in general, being increased in sick cats, and in particular in cats with FIP, in both the session of tests. Moreover, the magnitude of the differences between sick and clinically healthy cats was in all sessions higher than the intra- and inter-assay imprecision of the test, suggesting that differences between groups do not depend on the intrinsic variability of the test. As stated above, the effect of storage and freezing has not been investigated in this study. However, samples with long storage were present in all the groups and therefore the effect of freezing, if any, may have potentially affected the results of all the groups in the same manner. However, despite this possible limitation, the ROC curve analysis confirmed that CHIT1 activity had a good discriminating power to identify sick cats. The definition of diagnostic thresholds was out of the scope of the current preliminary study, as well as the establishment of the reference intervals for healthy cats, which requires higher numbers of observations and a well-defined diagnostic approach [18]. However, these issues deserve to be further investigated in future studies. When interpreting results of sick cats it should be considered that although the composition of the three groups in terms of breeds was similar, the median age of the group of cats with diseases other than FIP was significantly higher than that of cats with FIP and of clinically healthy cats, and the majority of cats in this group were males or castrated males, while in the other groups the proportion of male and female cats was similar. It is reported that CHIT1, chitinase-3-like-1 protein (CHI3L1) and chitinase-3-like 2 (CHI3L2) increase with age in humans [20,21,22,23]. Data regarding the correlation of CHIT1 with sex are lacking, while the activity of CHI3L1 is reported to be higher in females [20]. Therefore, the possible presence of age-, gender- or breed-related differences in serum CHIT1 activity in cats also merits further investigation in the future. However, it is unlikely that possible gender- or breed-related peculiarities in CHIT1 activity may have induced the significant difference recorded between healthy cats and cats with FIP, since this latter group had a similar distribution of age and gender compared with healthy cats. Moreover, the magnitude of the increased CHIT1 activity in the whole group of sick cats was so high as to be most likely the consequence of the pathological condition than a simple effect of age- or gender-related variability. Therefore, these results are consistent with the role of this enzyme in inflammation, which may account for an increased release of CHIT1 in blood [6]. Another finding consistent with a possible direct role of inflammation in the increases of CHIT1 recorded in this study is the highest value recorded in cats with FIP, a disease in which the activation of innate immunity and the recruitment of macrophages in inflammatory sites, as well as their activation, are particularly intense [24,25,26,27]. Ultimately, the results of neutrophil counts also confirm the presence of inflammation in sick cats, although neutrophilia is more severe in non-FIP cats than in FIP cats. Conversely, monocytes were not increased in sick cats compared with controls, and monocyte counts were not correlated with CHIT1 activity. This is not surprising since CHIT1 is released by activated macrophages and therefore not necessarily associated with an increase in circulating monocytes. Further studies are needed to understand which cell type is responsible for the increased CHIT1 activity recorded in sick cats, since the design of the current study does not allow the investigation of this hypothesis. For example, in vitro studies on CHIT1 expression by leukocytes isolated from the blood of cats with or without FIP or by feline monocyte-derived cells (e.g., *Felis catus* whole fetus 4–Fcwf-4–cells) incubated with the feline coronavirus may provide further insight about the CHIT1 responses induced by coronaviruses. Independent of these hypotheses on the source of CHIT 1, this enzyme may be proposed as a biomarker in cats with inflammation or FIP. The lack of statistical significance between cats with FIP and cats with other diseases may depend on the heterogeneity in this latter group, which was composed either by cats with diseases with a strong inflammatory pathogenesis and with clinical presentation potentially consistent with FIP (e.g., cholangitis, septic effusions) or cats with diseases that may or may not be confused with FIP at clinical presentation. These latter cats were included in order to assess whether CHIT1 activity may be influenced by a disease status in general or if only systemic and severe signs may be responsible for changes in CHIT1 activity. In these cats, however, the inflammatory component was moderate or secondary to other primary processes (e.g., cardiogenic effusions, allergic reactions, trauma, neoplasia). This heterogeneity, coupled with the high dispersion of data in the group of FIP cats, likely depending on the severity of the clinical presentation and of the associated inflammatory reaction, determined a partial overlapping between the groups of cats with and without FIP, and may also account for the lack of differences between cats with wet or dry FIP, despite effusive FIP usually being characterized by a more severe activation of innate immunity compared with dry FIP [27]. Moreover, the number of cats with dry FIP was very low and this may have contributed to masking the possible presence of significant differences between the two forms of the disease. Additionally, due to the overlapping mentioned above, the AUCs of the ROC curves designed by comparing cats with FIP to a single group composed of cats with diseases other than FIP were lower than those obtained by comparing sick vs. healthy cats. This suggests that CHIT1 alone cannot differentiate cats with FIP from cats with other diseases, especially in routine practice, where the analyte is measured for diagnostic purposes only in sick cats with clinical signs potentially consistent with FIP and not in clinically healthy cats, as in the dataset used to design the ROC curve in this study. As stated previously, however, the definition of diagnostic thresholds needs to be based on a higher caseload and on a different study design. Additionally, it may be considered that the diagnosis of FIP is usually based on a combination of clinical and laboratory tests aimed to determine a pre-test probability of disease before running confirmatory tests. From this perspective, based on the positive likelihood ratio higher than 4.00 recorded in the current study, increased CHIT1 activity may be a powerful support to achieve a conclusive diagnosis of FIP, once a high pre-test probability of FIP has been formulated based on anamnestic information, clinical findings and routine laboratory testing, as demonstrated in the past for other inflammatory markers [28]. Additionally, the design of this study does not allow the investigation of whether the magnitude of CHIT1 may also have a prognostic role in cats treated for FIP, since antiviral treatments that have been shown to be effective for FIP [29] cannot be prescribed or administered in our country. Therefore, this aspect also deserves to be further exploited through longitudinal studies in the future.

## 5. Conclusions

In conclusion, this preliminary study demonstrates that CHIT1 activity increases during diseases, especially in those with an inflammatory pathogenesis, and in particular in FIP, as the fluorimetric method employed in this study was precise and accurate enough to provide reliable results. However, some additional analytical and biological aspects are still to be elucidated before recommending the wide-scale use of this method in feline practice. In particular, future studies should be addressed to clarify whether long term storage or interferents may influence the measurement of CHIT1 activity, to establish feline reference intervals for this analyte (including any possible variation due to breed, gender and age) and to complete the assessment of its diagnostic or prognostic role in cats with inflammation, including the definition of diagnostic thresholds, through an increase in the caseload and a more standardized selection of feline patients affected by inflammatory conditions or by FIP, and possibly to monitor CHIT1 responses of FIP cats during the follow up, to investigate the possible prognostic usefulness of this analyte.

## Figures and Tables

**Table 1:** Demographic information about the cats included in the three study groups. Information regarding the clinical presentation, the diagnostic approach followed to group the cats, the final diagnoses and the number of neutrophils and monocytes in blood are also reported.

Group	Clinically Healthy (n = 17)	FIP (n = 19)	Non FIP (n = 20)
Breed	DSH (n = 13); British Shorthair, Maine Coon, Persian, Ragdoll (n = 1 each)	DSH (n = 18); Ragdoll (n = 1)	DSH (n = 19), Maine Coon (n = 1)
Sex	7 F, 2 sF, 5 M, 3 cM	3 F, 3 sF, 10 m, 3 cM	2 F, 8 sF, 2 M, 8 cM
Age range (median)	5 m–10 y (16 m)	4 m–5 y (14 m)	5 m–16 y (6.4 y)
Symptoms	None	Effusions, possibly associated with icterus, fever, ocular or neurological signs (n = 14); fever, neurological signs or abdominal masses (n = 5)	Symptoms potentially consistent with FIP (effusions, possibly associated with fever and/or jaundice) (n = 13), symptoms not consistent with FIP (e.g., cutaneous lesions, gastrointestinal signs, polytrauma (n = 7)
Diagnostic method	Clinical examination, hematology and clinical chemistry	Necropsy (n = 4); necropsy and immunohistochemistry (n = 6); positive PCR on CSF (n = 2); positive PCR on the effusion (n = 2); clinico-pathological changes on serum and effusions (n = 5) *	Diagnostic imaging, hematology and clinical chemistry, analysis of the effusions, when present, response to treatments, and/or histological analysis of samples collected post mortem or in vivo
Final diagnosis	None	Wet FIP (n = 14); dry FIP (n = 5)	Septic effusion (n = 4); cholangiohepatitis (n = 4); hyperproteinemia due to multiple myeloma (n = 2); trauma (n = 2); cardiopathy (n = 1); gastrointestinal eosinophilic sclerosing fibroplasia (n = 1); lymphoma (n = 1); cutaneous abscess (n = 1); follicular cyst (n = 1); lymphoplasmocytic enteritis and FIV (n = 1); gastroenteritis (n = 1); systemic allergic reaction (n = 1)
Blood neutrophils (×10³/µL) **	6.02 ± 2.63 (5.66)	9.60 ± 6.98 (8.57)	15.07 ± 14.7 ^†^ (11.03)
Blood monocytes (×10³/µL) **	0.27 ± 0.18 (0.23)	0.34 ± 0.47 (0.16)	0.73 ± 0.95 (0.45)

* These 5 cats had normocytic normochromic non-regenerative anemia, lymphopenia, hyperproteinemia with inverted A:G ratio and polyclonal gammopathy in blood and the analysis of the effusions was consistent with FIP (non-degenerated neutrophils, macrophages, lymphocytes and a granular proteinaceous background were found in cytology, and the delta total nucleated cell count measured on the effusions [11,12] was >1.7). ** Results are reported as mean ± standard deviation. The median value is reported in brackets. The numbers of samples with neutrophil and monocyte counts available were 14 (clinically healthy), 17 (FIP cats) and 19 (non FIP cats). ^†^ *p* < 0.01 vs. healthy.

**Table 2:** Results obtained in cats with FIP (n = 19), in cats with diseases other than FIP (n = 20) and in clinically healthy cats (n = 17).

Test Session	FIP	Non-FIP	Healthy	*p*
1	39.4 ± 60.4 (20.8) ^†^ 7.2–35.7	29.6 ± 37.7 (13.6) 6.8–42.9	14.5 ± 24.3 (6.4) 4.9–12.2	0.018
2	49.1 ± 101.7 (14.2) ^††^ 5.7–19.3	21.8 ± 32.7 (8.1) 5.1–26.6	9.8 ± 13.3 (4.9) 4.2–8.2	0.024

Results are reported as mean ± standard deviation, median (between parenthesis) and I–III interquartile range. Values are expressed in µg/mL. Results of statistical analysis are also reported. ^†^ = *p* < 0.037 vs. healthy; ^††^ = *p* < 0.028 vs. healthy.

**Table 3:** Results obtained in cats with dry FIP (n = 5) and in cats with wet FIP (n = 14).

Test Session	Dry FIP	Wet FIP	*p*
1	20.6 ± 14.9 (25.0) 5.2–33.2	46.1 ± 69.3 (18.7) 8.7–55.0	>0.005
2	16.8 ± 2.0 (16.6) 15.5–18.2	60.6 ± 1.17 (10.4) 4.9–45.0	>0.005

Results are reported as mean ± standard deviation, median (between parenthesis) and I–III interquartile range. Values are expressed in µg/mL. Results of statistical analyses are also reported.

**Table 4:** Summary of the diagnostic performances of CHIT1 activity to support a diagnosis of disease or of FIP in sessions 1 and 2.

Groups	Test Session	AUC (95% CI)	Max Youden Index	Highest LR+	100% Spec
Sick vs. Healthy	1	0.73 (0.59–0.88)	0.431 (8.31 µg/mL) Sens 72.5; Spec 70.6	5.10 (31.46 µg/mL)	105.29 µg/mL
	2	0.64 (0.48–0.80)	0.342 (15.16 µg/mL) Sens 63.2; Spec 71.1	3.83 (23.86 µg/mL)	162.39 µg/mL
FIP vs. non-FIP	1	0.72 (0.57–0.87)	0.406 (6.43 µg/mL) Sens 70.0; Spec 70.6	4.00 (105.23 µg/mL)	57.87 µg/mL
	2	0.67 (0.51–0.83)	0.368 (8.39 µg/mL) Sens 68.4; Spec 68.4	6.00 (61.00 µg/mL)	141.61 µg/mL

AUC = area under the curve; CI = confidence interval; LR+ = positive likelihood ratio; spec = specificity.

## Data Availability

The data presented in this study are available on request from the corresponding author.
